# Modified version of unified multiple system atrophy rating scale for remote video-based assessments

**DOI:** 10.1038/s41531-023-00590-1

**Published:** 2023-10-27

**Authors:** Yi Xiao, Lingyu Zhang, Qianqian Wei, Ruwei Ou, Yanbing Hou, Kuncheng Liu, Junyu Lin, Tianmi Yang, Jiang Qirui, Huifang Shang

**Affiliations:** 1 Department of Neurology, Rare Disease Center, Laboratory of Neurodegenerative Disorders, National Clinical Research Center for Geriatric, West China Hospital, Sichuan University, Chengdu, Sichuan China; 2 Health Management Center, West China Hospital of Sichuan University, Chengdu, China

**Keywords:** Neurodegenerative diseases, Medical research

## Abstract

We modified the original Unified Multiple System Atrophy Rating Scale (UMSARS) for remote video-based visits by excluding ocular motor dysfunction, increased tone, and body sway, resulting in a 23-item UMSARS (mUMSARS-23). The mUMSARS-23 demonstrated excellent reliability and strong validity when compared to the original scale, making it a promising tool for conducting video-based virtual assessments in patients with multiple system atrophy.

Multiple system atrophy (MSA) is a rapidly progressive neurodegenerative disorder featuring severe Parkinsonian disorders, ataxia, and autonomic dysfunction^1,2^. The emergence of the coronavirus disease 2019 pandemic increased the need for remote health services and telehealth-promoted studies^3^. Compared to in-person visits, remote visits are more cost-effective and time-efficient for both patients and trial sponsors^3^. Furthermore, the convenience of virtual visits can enhance the motivation of participants to take part in clinical trials^4^.

A modified version of the Unified Parkinson’s Disease Rating Scale (UPDRS) has been demonstrated to possess excellent reliability and validity^4,5^. Similarly, a modified Unified Huntington’s Disease Rating Scale used in virtual visits was found to have excellent reliability with the standard scale used in in-person visits^6^. A reliable and valid modified Progressive Supranuclear Palsy Rating Scale (PSPRS) for virtual assessment has been recently developed to facilitate virtual assessments^7^. To support the virtual assessments of patients with MSA, we aimed to develop a modified version of the Unified Multiple System Atrophy Rating Scale (UMSARS). Data from a longitudinal cohort was used to evaluate the cross-sectional and longitudinal agreement between modified UMSARS and standard UMSARS.

There were 432 patients with MSA included in the current study (Table 1). A total of 332 patients had the 1-year interview and UMSARS scores were available. The mean annual change of scores was 10.8 (standard deviation: 9.37) for UMSARS and 9.5 (standard deviation: 8.33) for mUMSARS-23. The intraclass correlation coefficient (ICC) between mUMSARS-23 and UMSARS was 0.995 at baseline, 0.995 at the 1-year visit, and 0.985 for the change of baseline and 1-year visit. Excellent agreements were reached in cross-sectional and longitudinal analyses. The scatter plots, best-fit line, and 95% prediction interval about the best-fit line at baseline and 1-year visits were presented in Supplementary Fig. 1. Pearson’s correlation analysis also showed strong concurrent validity between UMSARS and mUMSARS-23 at baseline (*r* = 0.995, *p* < 0.001), 1-year visit (*r* = 0.996, *p* < 0.001), and longitudinally (*r* = 0.985, *p* < 0.001). In the simulated video-based assessment, we included 50 MSA patients (Table 1). We found that the simulation assessment of mUMSARS-23 also had an excellent agreement (ICC: 0.964) and strong concurrent validity (*r* = 0.969, *p* < 0.001).

**Table 1:** Demographics of patients with MSA.

Demographic	MSA in developing cohort (*n* = 432)	MSA in simulation assessment cohort (*n* = 50)
Age	59.92 (8.16)	62.02 (8.23)
Age of onset	57.56 (8.09)	59.43 (8.00)
Male	220 (50.9%)	26 (52%)
MSA-P	217 (50.2%)	36 (72%)
Disease duration	2.33 (1.45)	2.58 (1.91)
UMSARS	33.36 (13.96)	26.90 (15.78)
mUMSARS-23	29.82 (12.55)	24.92 (15.00)
SOH	180 (41.7%)	11 (22%)
UMSARS part IV		
1	132 (30.6%)	25 (50%)
2	184 (42.6%)	18 (36%)
3	67 (15.5%)	2 (4%)
4	44 (10.2%)	2 (4%)
5	5 (1.2%)	3 (6%)

Data were presented as mean (standard deviation) or number (percent).

*MSA* multiple system atrophy, *MSA-P* MSA-Parkinsonism, *UMSARS* the Unified Multiple System Atrophy Rating Scale, *mUMSARS-23* modified 23 items of UMSARS, *SOH* significant orthostatic hypotension.

For the survival analysis, there were 157 patients died during the follow-up. The Cox regression model showed that a higher mUMSARS-23 score at baseline was associated with an increased risk of death. (Table 2) At the 1-year visit, 313 patients out of 332 patients had consistent disease progression direction between UMSARS and mUMSARS-23 assessments and the total percent agreement was 94.3%. The kappa estimate was 0.733 (standard error: 0.058, p < 0.001), indicating a substantial agreement between the UMSARS and mUMSARS-23.

**Table 2:** Survival analysis in patients with MSA using UMSARS or mUMSARS-23.

	HR (95% CI)	*p*-value
Model 1		
Age	1.014 (0.994–1.035)	0.182
Male	1.249 (0.907–1.719)	0.173
Disease duration	0.619 (0.536–0.714)	<0.001*
Subtype (MSA-P)	1.294 (0.912–1.837)	0.149
SOH	1.570 (1.127–2.188)	0.008*
UMSARS	1.043 (1.032–1.054)	<0.001*
Model 2		
Age	1.013 (0.992–1.034)	0.230
Male	1.244 (0.904–1.712)	0.181
Disease duration	0.616 (0.533–0.712)	<0.001*
Subtype (MSA-P)	1.309 (0.922–1.859)	0.132
SOH	1.553 (1.115–2.163)	0.009*
mUMSARS-23	1.049 (1.037–1.061)	<0.001*

*HR* hazard ratio, *CI* confidence interval, *MSA* multiple system atrophy, *MSA-P* MSA-parkinsonism, *UMSARS* the Unified Multiple System Atrophy Rating Scale, *mUMSARS-23* modified 23 items of UMSARS, *SOH* significant orthostatic hypotension.

*Significant at level 0.05.

The present study created a modified version of UMSARS (mUMSARS-23) and assessed the reliability and validity of the mUMSARS-23 in cross-sectional and longitudinal evaluations. The results demonstrated excellent reliability, as indicated by the high ICC values, and strong correlations between UMSARS and mUMSARS-23 scores at baseline, the 1-year visit, and for annual changes. Additionally, the successful video-based assessment using mUMSARS-23 provided further support for the reliability and validity of mUMSARS-23. Survival analysis revealed a significant association between higher baseline mUMSARS-23 scores and an increased risk of mortality, confirming the validity of the modified scale. Furthermore, a substantial agreement between mUMSARS-23 and UMSARS was demonstrated by the high percentage of patients with consistent disease progression directions and the kappa coefficient.

Our findings revealed a notable association between significant orthostatic hypotension and a heightened risk of mortality, aligning with the outcomes of a meta-analysis study^8^. This link suggested that orthostatic hypotension may indicate more severe dysfunction of the autonomic nervous system and overall disease severity, thereby contributing to an increased risk of mortality^8^. Notably, respiratory infection emerged as the most prevalent cause of death, followed by sudden death, and nutritional disorders. These causes of death were found to be highly related to MSA, underscoring the significant impact of the disease on patients’ health outcomes^9^. The observed death rate in our study (36.3%) closely mirrored the reported rates in previous studies, which ranged from 30.7% to 40%^10,11^. These findings provide valuable insights into the impact of orthostatic hypotension on mortality risk and the most common causes of death in patients with MSA.

A less modified version of UMSARS incorporating body sway might be perceived as having similar validity compared to mUMSARS-23, as the closer it remains to the original version, the higher its potential validity. Nevertheless, concerns arise regarding the remote assessment of body sway, which could lead to inaccurate results due to varying strengths or incorrect guidance from untrained performers, such as caregivers or patients’ relatives. Considering the potential risk associated with incorrect execution of the body sway test by remote performers, we decided to exclude it from mUMSARS-23 to ensure the assessment’s accuracy and safety. (For further discussion see Supplementary Discussion).

The strength of our study was the confirmation of mUMSARS-23s validity through longitudinal follow-up. This established a solid foundation for its utilization in cohort studies and clinical trials. The limitation of the current study was that the feasibility of mUMSARS-23 in remote virtual visits was not confirmed, and unexpected challenges might have arisen in clinical practice. To address this concern, we have conducted a video-based assessment to simulate the process of remote virtual visits. The results demonstrated good reliability and validity, supporting the use of mUMSARS-23 in video-based virtual visits.

In conclusion, we have successfully developed a modified version of UMSARS (mUMSARS-23) tailored for remote video-based visits. The mUMSARS-23 demonstrated excellent reliability and validity compared to UMSARS, making it a valuable and effective tool in telehealth for patients with MSA.

## Methods

### Patients

Patients who met the 2008 diagnostic criteria of probable MSA were recruited from the Department of Neurology, West China Hospital, Sichuan University from June 2011 to April 2021^12^. UMSARS score and other clinical characteristics were collected through face-to-face interviews conducted by experienced neurological doctors. All raters were trained and had excellent agreement in inter-rater reliability^13^. Significant orthostatic hypotension was defined as a drop in systolic blood pressure ≥30 mm Hg and/or diastolic blood pressure ≥15 mm Hg. Patients were invited for annual face-to-face follow-up visits and were followed up by telephone for survival. This study was approved by the Institutional Ethics Committee of West China Hospital. Written informed consent was obtained from all the participants of the study.

### Developing modified UMSARS (mUMSARS-23)

The score of UMSARS was the sum of part I and part II, with higher scores indicating a more severe disease. To adapt the scale for remote video-based assessment, we removed ocular motor dysfunction, increased tone, and body sway (questions 3, 6, and 13 from UMSARS part II), as the accurate evaluation in these contexts was challenging in remote assessment^14^. Considering the impact of internet speed and remote device management by patients or caregivers^3^, ocular motor dysfunction was removed to avoid any potential inaccuracies in the practical application of mUMSARS-23. Additionally, the implementation of body sway by non-medical caregivers with no professional training might inadvertently cause dangerous events, such as falls, so we removed this item from the modified scale. As a result, we created a modified version of UMSARS, mUMSARS-23, comprising 23 items from part I and part II.

### Simulation of video-based assessment for mUMSARS-23

After developing the mUMSARS-23, we tested its reliability and validity using a simulated remote video-based assessment at our center between June 2023 and July 2023. All the patients included in this part met the 2008 diagnostic criteria of probable or possible MSA^12^. During face-to-face interviews, HYB, LJY, and YTM raters conducted the face-to-face interview with the patients and recorded the evaluation of UMSARS Part I and Part II on videotape. Subsequently, JQR, without prior knowledge of the in-person assessment results, meticulously assessed the videotapes using mUMSARS-23 in a simulated evaluation. Then, ZLY analyzed the results of both face-to-face assessment (using UMSARS) and simulation assessment (using mUMSARS-23).

### Reliability analysis

First, we converted the raw score to a proportion of the maximum possible score of each scale, respectively. Cross-sectional reliability was assessed using the ICC of proportional scores of mUMSARS-23 and standard UMSARS at baseline and the 1-year visit. Longitudinal reliability was assessed by calculating the ICC of change of proportional scores between baseline and 1-year visit of two versions of scales. Excellent agreement was established as ICC greater than or equal to 0.81^15^.

### Validity analysis

We used Pearson’s correlation coefficients (r) between the proportional scores of standard UMSARS and mUMSARS-23 to assess the concurrent validity at baseline, 1-year visit, and longitudinally (change between baseline and 1-year visit). A strong concurrent validity was established with *r* > 0.80. Then, we assessed the criterion validity by survival analysis. Cox proportional hazards models were used for survival analysis and scores of mUMSARS-23 were included as a predictor of survival, adjusted for the age, sex, disease duration, diagnostic type, and baseline significant orthostatic hypotension.

### Kappa and percent agreement analysis

A higher score of UMSARS or mUMSARS-23 indicated a more severe disease. Patients were divided into remission/stabilization or deterioration according to the change of score from baseline to the 1-year. We used the kappa test to evaluate the agreement of individual disease progression directions between UMSARS and mUMSARS-23. Excellent agreement was considered as kappa estimates above 0.8 and substantial agreement was above 0.6^16^. The statistical significance was set as a *p*-value < 0.05. All the analyses were conducted using SPSS 26.0.

### Reporting summary

Further information on research design is available in the Nature Research Reporting Summary linked to this article.

### Supplementary information

supplementary file

reporting summary

## Data Availability

Anonymized data generated during the current study are available from the corresponding author on request from individuals affiliated with research or healthcare institutions. UMSARS and modified UMSARS are available from the International Parkinson and Movement Disorder Society (https://www.movementdisorders.org/).

## Code availability

The code used in the current study is available from the corresponding author.
