# Supplementary material for: Modified version of unified multiple system atrophy rating scale for remote video-based assessments
Source: NPJ Parkinsons Dis. 2023 Oct 27;9:147. doi: 10.1038/s41531-023-00590-1 (PMC10611709; doi:10.1038/s41531-023-00590-1)
Supplement: Supplementary file 1 — supplementary file [file 41531_2023_590_MOESM1_ESM.docx]

**Supplementary Discussion**

Deleting a controlled number of items can remain good reliability and validity of scales.^1-3^ According to Wills et.al, a modified PSPRS which has removed seven items from a total of 28 items had an excellent agreement with the original scale.^2^ However, when missing more than three items of the total 33 items of Part III of the Movement Disorder Society Unified Parkinson’s Disease Rating Scale, an accurate surrogate part score could not be obtained.^1^ In our study, we deleted three items from the total 26 items of UMSARS and found that the modified scale had an excellent agreement with the UMSARS. The ocular motor dysfunction and body sway were not sensitive to changes in the disease severity in MSA.^4^ Deleting these items can simplify the scale. An online registry and visit trial confirmed the feasibility and patient's satisfaction with virtual visits in patients with PD.^5^ Great correlation was observed in the patients-reported outcomes and a moderate correlation was found in motor assessment between remote and in-person assessment in patients with PD.^6^ However, with no data being available to date, the virtual trail of patients with MSA was expected to confirm the feasibility and validity of remote video-based visits. In addition, compared to other assessments used in remote visits, such as remote smart devices and artificial intelligence,^7^ a modified scale had lower technology and equipment barriers and the data from each research can be compared transversely.

1 Goetz, C. G. *et al.* Handling missing values in the MDS-UPDRS. *Movement disorders : official journal of the Movement Disorder Society* **30**, 1632-1638, doi:10.1002/mds.26153 (2015).

2 Wills, A. M. *et al.* A Modified Progressive Supranuclear Palsy Rating Scale for Virtual Assessments. *Movement disorders : official journal of the Movement Disorder Society*, doi:10.1002/mds.28991 (2022).

3 Abdolahi, A., Scoglio, N., Killoran, A., Dorsey, E. R. & Biglan, K. M. Potential reliability and validity of a modified version of the Unified Parkinson's Disease Rating Scale that could be administered remotely. *Parkinsonism & related disorders* **19**, 218-221, doi:10.1016/j.parkreldis.2012.10.008 (2013).

4 Krismer, F. *et al.* Sensitivity to Change and Patient-Centricity of the Unified Multiple System Atrophy Rating Scale Items: A Data-Driven Analysis. *Movement disorders : official journal of the Movement Disorder Society*, doi:10.1002/mds.28993 (2022).

5 Dorsey, E. R. *et al.* Feasibility of Virtual Research Visits in Fox Trial Finder. *Journal of Parkinsons Disease* **5**, 505-515, doi:10.3233/jpd-150549 (2015).

6 Tarolli, C. G. *et al.* Feasibility, Reliability, and Value of Remote Video-Based Trial Visits in Parkinson's Disease. *Journal of Parkinsons Disease* **10**, 1779-1786, doi:10.3233/jpd-202163 (2020).

7 Larson, D. N., Schneider, R. B. & Simuni, T. A New Era: The Growth of Video-Based Visits for Remote Management of Persons with Parkinson's Disease. *J Parkinsons Dis* **11**, S27-S34, doi:10.3233/JPD-202381 (2021).

**Supplementary Figure 1** Scatterplots for modified UMSARS versus standard motor UMSARS (A) at baseline, and (B) at 1-year visit. Solid lines represent the best-fit linear regression line and the dashed line represents the 95% prediction interval of the best-fit line. All the scores were presented as proportional scores.

UMSARS: the Unified Multiple System Atrophy Rating Scale.


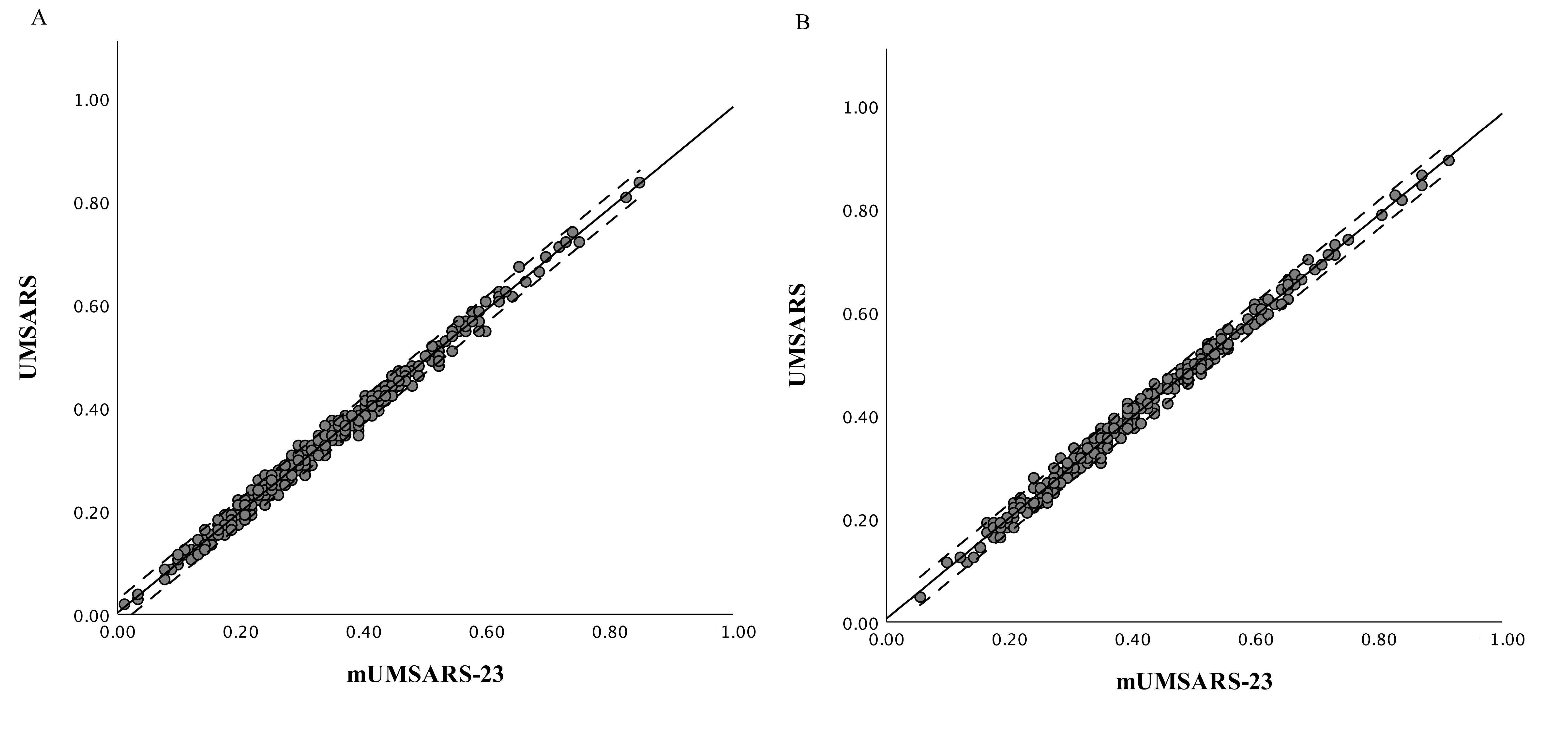


Supplementary Table 1 Cause of death in patients with MSA.

| Cause of death | Number of patients |
| --- | --- |
| Multiple organs dysfunction syndrome | 2 |
| Respiratory infection | 66 |
| Urinary tract infection | 10 |
| Choking | 7 |
| Pressure ulcers | 2 |
| Nutritional disorder | 14 |
| Suicide | 3 |
| Sudden death | 37 |
| Trauma from falls | 3 |
| Unclear | 12 |
| Surgical accident | 1 |

MSA: multiple system atrophy.

Supplementary References

**References**

1 Low, P. A. *et al.* Natural history of multiple system atrophy in the USA: a prospective cohort study. *The Lancet Neurology* **14**, 710-719, doi:10.1016/s1474-4422(15)00058-7 (2015).

2 Foubert-Samier, A. *et al.* Disease progression and prognostic factors in multiple system atrophy: A prospective cohort study. *Neurobiol Dis* **139**, 104813, doi:10.1016/j.nbd.2020.104813 (2020).

3 Larson, D. N., Schneider, R. B. & Simuni, T. A New Era: The Growth of Video-Based Visits for Remote Management of Persons with Parkinson's Disease. *J Parkinsons Dis* **11**, S27-S34, doi:10.3233/JPD-202381 (2021).

4 Tarolli, C. G. *et al.* Feasibility, Reliability, and Value of Remote Video-Based Trial Visits in Parkinson's Disease. *Journal of Parkinsons Disease* **10**, 1779-1786, doi:10.3233/jpd-202163 (2020).

5 Abdolahi, A., Scoglio, N., Killoran, A., Dorsey, E. R. & Biglan, K. M. Potential reliability and validity of a modified version of the Unified Parkinson's Disease Rating Scale that could be administered remotely. *Parkinsonism & related disorders* **19**, 218-221, doi:10.1016/j.parkreldis.2012.10.008 (2013).

6 Bull, M. T. *et al.* A Pilot Study of Virtual Visits in Huntington Disease. *Journal of Huntingtons Disease* **3**, 189-195, doi:10.3233/jhd-140102 (2014).

7 Wills, A. M. *et al.* A Modified Progressive Supranuclear Palsy Rating Scale for Virtual Assessments. *Movement disorders : official journal of the Movement Disorder Society*, doi:10.1002/mds.28991 (2022).

8 Gilman, S. *et al.* Second consensus statement on the diagnosis of multiple system atrophy. *Neurology* **71**, 670-676, doi:10.1212/01.wnl.0000324625.00404.15 (2008).

9 Zhang, L. *et al.* Causes of Death in Chinese Patients with Multiple System Atrophy. *Aging Dis* **9**, 102-108, doi:10.14336/AD.2017.0711 (2018).

10 Wenning, G. K. *et al.* Development and validation of the Unified Multiple System Atrophy Rating Scale (UMSARS). *Movement disorders : official journal of the Movement Disorder Society* **19**, 1391-1402, doi:10.1002/mds.20255 (2004).

11 Shrout, P. E. & Fleiss, J. L. Intraclass correlations: uses in assessing rater reliability. *Psychol Bull* **86**, 420-428, doi:10.1037//0033-2909.86.2.420 (1979).

12 Landis, J. R. & Koch, G. G. The measurement of observer agreement for categorical data. *Biometrics* **33**, 159-174 (1977).

13 Glasmacher, S. A., Leigh, P. N. & Saha, R. A. Predictors of survival in progressive supranuclear palsy and multiple system atrophy: a systematic review and meta-analysis. *Journal of neurology, neurosurgery, and psychiatry* **88**, 402-411, doi:10.1136/jnnp-2016-314956 (2017).

14 Riku, Y. *et al.* Non-motor multiple system atrophy associated with sudden death: pathological observations of autonomic nuclei. *Journal of neurology* **264**, 2249-2257, doi:10.1007/s00415-017-8604-y (2017).

15 Chelban, V. *et al.* Neurofilament light levels predict clinical progression and death in multiple system atrophy. *Brain : a journal of neurology* **145**, 4398-4408, doi:10.1093/brain/awac253 (2022).

16 Wenning, G. K. *et al.* The natural history of multiple system atrophy: a prospective European cohort study. *The Lancet Neurology* **12**, 264-274, doi:10.1016/s1474-4422(12)70327-7 (2013).
